# Chronic respiratory disorders due to aberrant innominate artery: a case series and critical review of the literature

**DOI:** 10.1186/s13052-023-01473-0

**Published:** 2023-07-22

**Authors:** Adele Corcione, Melissa Borrelli, Leonardo Radice, Oliviero Sacco, Michele Torre, Francesco Santoro, Gaetano Palma, Eleonora Acampora, Francesca Cillo, Pietro Salvati, Angelo Florio, Francesca Santamaria

**Affiliations:** 1 Departments of Translational Medical Sciences, Pediatric Pulmonology, Federico II University, Naples, Italy; 2 Departments of Advanced Biomedical Sciences, Radiology Unit, Federico II University, Naples, Italy; 3 Department of Pediatrics, Gaslini University Hospital, Genoa, Italy; 4 Pediatric Thoracic and Airway Surgery Unit, Gaslini University Hospital, Genoa, Italy; 5 Cardiac and Vascular Surgery Unit, G, Gaslini University Hospital, Genoa, Italy; 6 Departments of Advanced Biomedical Sciences, Pediatric Cardiac Surgery, Federico II University, Naples, Italy

**Keywords:** Aberrant innominate artery, Chronic dry cough, Recurrent respiratory infections, Recurrent pneumonia, Tracheal compression

## Abstract

**Background:**

Tracheal compression (TC) due to vascular anomalies is an uncommon, but potentially serious cause of chronic respiratory disease in childhood. Vascular slings are congenital malformations resulting from abnormal development of the great vessels; in this group of disorders the most prevalent entity is the aberrant innominate artery (AIA). Here we provide a report on diagnosis and treatment of AIA in nine children with unexplained chronic respiratory symptoms. We describe the cases, perform a literature review, and provide a discussion on the diagnostic workup and treatment that can help manage AIA.

**Methods:**

Clinical history, diagnostic procedures and treatment before and after the AIA diagnosis were retrospectively reviewed in nine children (5 boys and 4 girls), who were referred for recurrent-to-chronic respiratory manifestations over 10 years (2012–2022). We performed a comprehensive report on the ongoing clinical course and treatment as well as an electronic literature search on the topic.

**Results:**

Diagnoses at referral, before AIA was identified, were chronic dry barking cough associated with recurrent pneumonia (*n* = 8, 89%), lobar/segmental atelectasis (*n* = 3, 33%), atopic/non atopic asthma (*n* = 3, 33%); pneumomediastinum with subcutaneous emphysema complicated the clinical course in one case. When referred to our Unit, all patients had been previously treated with repeated antibiotic courses (*n* = 9, 100%), alone (*n* = 6, 67%) or combined with prolonged antiasthma medications (*n* = 3, 33%) and/or daily chest physiotherapy (*n* = 2, 22%), but reported only partial clinical benefit. Median ages at symptom onset and at AIA diagnosis were 1.5 [0.08–13] and 6 [4–14] years, respectively, with a relevant delay in the definitive diagnosis (4.5 years). Tracheal stenosis at computed tomography (CT) was ≥ 51% in 4/9 cases and ≤ 50% in the remaining 5 subjects. Airway endoscopy was performed in 4 cases with CT evidence of tracheal stenosis ≥ 51% and confirmed CT findings. In these 4 cases, the decision of surgery was made based on endoscopy and CT findings combined with persistence of clinical symptoms despite medical treatment. The remaining 5 children were managed conservatively.

**Conclusions:**

TC caused by AIA may be responsible for unexplained chronic respiratory disease in childhood. Early diagnosis of AIA can decrease the use of expensive investigations or unsuccessful treatments, reduce disease morbidity, and accelerate the path toward a proper treatment.

## Introduction

Upper and lower airways chronic diseases are increasing in prevalence everywhere, particularly among children and elderly people. In children and adolescents, chronic cough can be a major manifestation of several recurrent-to-chronic respiratory diseases [1]. In many cases the etiology of the disease remains elusive, and a misidentification of the underlying disorder results in failure to start effective treatment [2].

Tracheal compression (TC) from congenital vascular anomalies is an uncommon, but potentially serious, cause of chronic respiratory disease in childhood. Although compression of the upper airway is typically associated with vascular abnormalities, other conditions may be responsible for TC. Among these, abnormal thoracic configuration as pectus excavatum, narrow chest diameter, scoliosis, rib anomalies and small left hemithorax have been described [3]. TC can also occur by space occupying lesions such as anterior mediastinal masses like Hodgkin or non-Hodgkin lymphomas, neurofibromas occurring in neurofibromatosis [4, 5], goiter, and cysts [6]. A case of airway compression by a large osteochondroma, arising from chest wall and sternum as a part of hereditary multiple exostoses, has also been described [7].

Stridor with cyanosis and apnea may be a presenting feature of TC in infancy, while incessant dry croup-like “seal-bark” cough, which sometimes is misdiagnosed as asthma, is commonly reported in older children [8]. Vascular slings are congenital malformations resulting from abnormal development of the great vessels; in this group of disorders the most prevalent entity is the aberrant innominate artery (AIA). Although accurate epidemiological data are lacking, AIA is an extremely common congenital vascular disorder, with 3% incidence [9, 10]. In normal individuals, the innominate artery crosses the trachea anteriorly after arising from the left side of the aortic arch. In AIA patients, the origin of innominate artery from the left side of the aortic arch is more distal than in normal conditions, thus causing TC. Severity of airway symptoms depends on the rate of external compression of the airway lumen and reflects secondary tracheomalacia. The patient’s ability to clear secretions from the distal airways is often impaired, and recurrent respiratory infections (RRI) may occur [11]. Importantly, a late AIA diagnosis increases the risk of prolonged damage to the airways [10]. Moreover, a severe TC demands a surgical repair [12], but patients should be thoroughly investigated before deciding whether operative or conservative treatment should be performed.

We herein retrospectively describe a case series of children with recurrent-to-chronic respiratory manifestations who underwent repeated investigations and ineffective therapies before TC due to AIA was demonstrated. We also carried out an electronic keyword-based literature search for English articles published on this topic that could improve the diagnostic path and influence the choice of treatment in children with AIA.

## Methods

This is a retrospective case series of 9 pediatric patients referred to our Unit for recurrent-to-chronic respiratory symptoms. For each patient, we described the initial clinical manifestations and the winding path prior the diagnosis of AIA was confirmed. Finally, once AIA was diagnosis, we reported the treatment choice (*i.e.*, operative, or conservative) and commented on the current clinical course. We also carried out an electronic keyword-based literature search for English original articles and/or case series ever published on this topic up to December 31, 2022, in the Scopus, Web of Science, PubMed, and MEDLINE databases. Studies conducted exclusively on adults and anecdotal single case reports were excluded. The terms “aberrant innominate artery” AND dry cough OR bark cough OR barking cough OR chronic cough OR recurrent respiratory infections OR recurrent pneumonia OR diagnosis OR treatment OR complications were used as keywords in combination. The identified studies were further evaluated to select only relevant literature, and, in addition, a manual search was conducted to evaluate references from review articles.

## Results

### Case series

The charts of 9 children (5 boys; 4 girls) admitted to the Pediatric Pulmonology, Department of Translational Medical Sciences, Federico II University, Naples, over a 10-year period (2012–2022) were reviewed. All were living in Campania (Southern Italy). Table 1 summarizes the clinical manifestations and the history of the study population, including the diagnostic work-up and treatment either before or after the diagnosis of AIA. The therapeutic approach to AIA, conservative or operative, and the current outcome are also reported. Diagnoses at referral were chronic dry cough (100% of the cases) that was associated with recurrent pneumonia (*n* = 8; 89%) or lobar/segmental atelectasis (*n* = 3; 33%) or atopic/non atopic asthma (*n* = 3, 33%), and included pneumomediastinum as additional complication in one case (11%). Treatment at referral prior to AIA diagnosis included prolonged repeated antibiotic courses (*n* = 9; 100%), alone or combined with prolonged antiasthma medications (*n* = 3; 33%) and/or daily chest physiotherapy in cases with recurrent pneumonia and lobar atelectasis (*n* = 2; 22%), with only partial clinical benefit. Median age at symptoms onset and at AIA diagnosis were 1.5 [range, 0.08–13] and 6 [range, 4–14] years, respectively, with a noticeable delay in the definitive diagnosis (4.5 years).

**Table 1:** Clinical characteristics, diagnostic work-up and treatment of the study population

	Case 1 (male)	Case 2 (male)	Case 3 (female)	Case 4 (female)	Case 5 (male)	Case 6 (male)	Case 7 (male)	Case 8 (female)	Case 9 (female)
Diagnosis at referral	Chronic dry cough Atopic asthma RP ML atelectasis	Chronic dry cough RP	Chronic dry cough RP ML atelectasis	Chronic dry cough RP Segmental atelectasis	Chronic dry cough RP	Chronic dry cough RP	Chronic dry cough Non atopic asthma	Chronic dry cough RP	Chronic dry cough Atopic asthma RP PM
Treatment at referral	Antibiotics Chest PT Antiasthma	Antibiotics	Antibiotics Chest PT	Antibiotics	Antibiotics	Antibiotics	Antibiotics Antiasthma	Antibiotics	Antibiotics Antiasthma
Age at onset (*years*)	0.6	1.5	3	1	2	0.08	13	0.4	4
Age at AIA diagnosis *(years)*	6	4	7	4	5	8	13	6	14
Whole diagnostic work-up^a^	Spirometry; FeNO; nNO/NB; ST/CFTR; sIg/vaccines response; Chest X-ray/CT AE	NB; ST/CFTR; sIg/vaccines response; Chest X-ray/CT	Spirometry; FeNO; nNO/NB; ST/CFTR; sIg/vaccines response; Chest X-ray/CT AE	NB; ST/CFTR; sIg/vaccines response; Chest X-ray/CT AE	NB; ST/CFTR; sIg/vaccines response; Chest X-ray/CT MII-pH	Spirometry; FeNO; nNO/NB; ST/CFTR; sIg/vaccines response; Chest X-ray/CT	Spirometry FeNO; nNO ST/CFTR;sIg/vaccines response; MII-pH Chest MRI	Spirometry; FeNO; nNO/NB; ST/CFTR; sIg/vaccines response; Chest X-ray/CT	Spirometry; FeNO; nNO/NB; ST/CFTR; sIg/vaccines response; Chest X-ray/CT MII-pH AE
AIA treatment	Aortopexy	Conservative	Aortopexy	Tracheopexy	Conservative	Conservative	Conservative	Conservative	Tracheopexy
Current symptoms	None	None	None	None	None	None	None	None	None

*Abbreviations*: *RP* recurrent pneumonia, *ML* middle lobe, *PM* pneumomediastinum, *AIA* aberrant innominate artery**,** *AE* airway endoscopy, *MII-pH* multichannel intraluminal impedance-pH, *PT* physiotherapy, *FeNO* fractional exhaled nitric oxide, *nNO* nasal nitric oxide, *NB* nasal brush, *ST* sweat test, CFTR cystic fibrosis transmembrane regulator, *sIg* serum immunoglobulins levels, *CT* computed tomography, *MRI* Magnetic Resonance Imaging

^a^Including findings collected over either pre- or post-AIA diagnosis period

Once admitted at our Unit, an internal diagnostic protocol was applied to investigate children with chronic cough and RRI. Sweat chloride test and CFTR analysis; nasal nitric oxide plus transmission electron microscopy and beat analysis of ciliary ultrastructure and motility, respectively, on nasal brushing; and immune status assessment (including serum total immunoglobulins levels and response to immunizations) for ruling out cystic fibrosis (CF), primary ciliary dyskinesia (PCD) and primary immunodeficiency (PID), respectively, were negative or normal.

All cases underwent routine echocardiography as a standard of care to evaluate suspected vascular abnormality and myocardial function, and results excluded a coexisting heart disease. Multichannel intraluminal impedance-pH monitoring, obtained in cases of reported gastrointestinal disturbances (such as troublesome heartburn and/or vomiting) (cases 2 and 7), ruled out gastroesophageal reflux disease. A computed tomography (CT) scan with and without contrast medium was performed in all patients without general anesthesia to confirm the diagnosis and measure the percentage of tracheal obstruction. Grading system of tracheal stenosis was used to stratify tracheal stenosis in four grades (grade I: stenosis up to 50%; grade II: stenosis between 51 and 70%; grade III: stenosis > 70%; grade IV: no lumen visualized at the narrowest point) [13]. Image analysis was performed on an offline workstation (Multimodality Workplace, Toshiba Healthcare). CT tracheal stenosis was ≥ 51% (grade II) in 4 cases (44%) and up to 50% (grade I) in the remaining 5 subjects (55%). We chose to perform airway endoscopy (AE) only in those 4 patients with CT evidence of tracheal stenosis ≥ 51%. Based on tracheal compression degree and on severity of clinical course, cases were either managed conservatively (and monitored in follow-ups) or surgically treated. Surgery was performed if tracheal stenosis was ≥ 51% and was associated with persistent clinical symptoms unresponsive to medical or supportive medical treatment (*i.e.*, recurrent pneumonia associated or not with complications such as lobar atelectasis and/or chronic asthma).

We herein briefly describe the clinical course either prior or after the diagnosis of AIA of two cases who were surgically or conservatively treated.

### Case 1

**Fig. 1:**
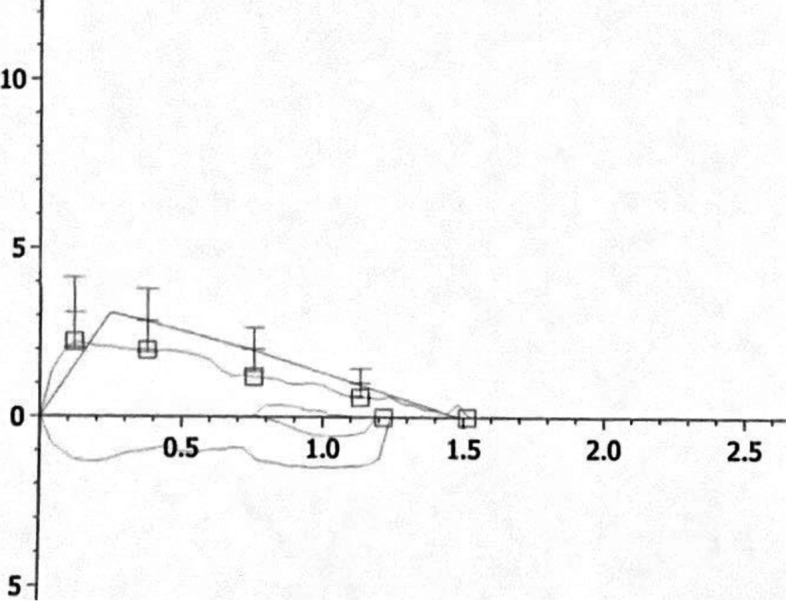
The flow volume loop from case 1 shows a plateau in both expiratory and inspiratory phase, suggesting fixed intrathoracic obstruction

**Fig. 2:**
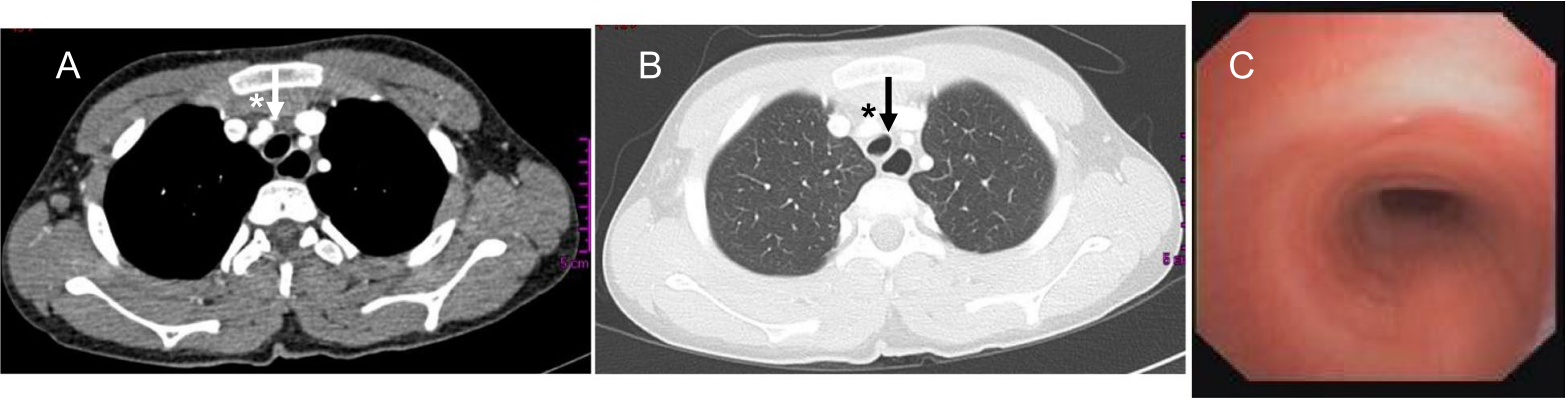
The CT scans from case 1 show (**A**) the anomalous course of the innominate artery (as indicated by the white arrow and the white asterisk) (**B**) an anterior compression on the right tracheal wall that induces a reduction of the tracheal caliber (as indicated by the black arrow and the black asterisk) (**C**) the endoscopic view that confirms the tracheal compression

The boy was born at term after an uneventful pregnancy. Family history revealed season allergic rhinitis in the father. Environmental history excluded parental cigarette smoking inside or outside home. Clinical well-being was reported from birth up to age 8 months, when recurrent preschool wheezing episodes due to viral infection associated with dry, barking cough were reported. At 4-year-old age, atopic bronchial asthma was diagnosed (skin tests positive to *Dermatophagoides spp* and *Parietaria*). Maintenance treatment with inhaled corticosteroids (ICS) combined with rescue inhaled albuterol was prescribed, with partial clinical benefit. At age 18 months, recurrent upper and lower respiratory tract infections started, and from 2- to 4-year-old age 3 episodes of pneumonia were documented at chest X-ray, which required hospitalization and antibiotic treatment. Middle lobe atelectasis was documented at chest CT without contrast media, demanding repeated antibiotic treatments and daily chest physiotherapy. However, dry barking cough frequently recurred at any upper or lower respiratory tract infection. At age 6, a more severe pneumonia event required hospital admission. Once ruled out CF, PCD and PID, we obtained a spirometry that showed a plateau in both expiratory and inspiratory phase (Fig. 1). The chest CT with contrast showed the anomalous course of the innominate artery and an anterior compression on the right tracheal wall inducing a tracheal stenosis quantified as grade II **(**Fig. 2A, B). Bronchoscopy showed an extrinsic pulsatile compression of the anterior wall of the trachea (between the middle and the distal part) with a lumen reduction > 50% (Fig. 2C). After cardiothoracic surgical consultation, aortopexy was made without any complication. The follow-up showed complete disappearance of the symptoms.

### Case 2

The boy was born preterm at 36 weeks due to premature rupture of membranes, but neither birth complications nor need for ventilatory support were reported. Family history was negative for allergy and there was no passive exposure to smoke cigarette at home. The child was healthy until the age of 18 months, when pneumonia with lower right lobe consolidation was documented and oral antibiotics were administered. Since then, recurrent upper and lower airways infections with persistent dry, barking cough occurred. At least 2 episodes of pneumonia localized at the right lower lobe were reported. At age 4 years, the patient was referred to our Unit, and we ruled out atopy, PID, CF and PCD. The chest CT with contrast medium showed the anomalous course of the innominate artery with mild anterolateral compression on the right tracheal wall (grade I tracheal stenosis). Given the slight extent of the restriction of the tracheal lumen, we decided not to perform the airway endoscopy. At moment, the patient (age 5 years) is in good clinical conditions. As there was a progressive, substantial reduction of the frequency of the airways infections and cough has been rarely reported in the last 2 years, we decided not to refer the boy to the surgeon, but rather continue the monitoring of the clinical course.

### Review of the literature

Table 2 summarizes the main findings from 20 original articles reporting data on 2166 patients with several vascular anomalies, including 1092 patients with AIA. Patients were followed at 5 EU, 7 US and 1 Canada centers. Of all studies (3 prospective [14–16] and 17 retrospective [10–12, 17–30]), the oldest was dated 1963 [17] and the most recent was published in 2015 [10]. The patients’ age range was 0 to 17 years. Prevalence of presenting features was extremely variable, likely because different criteria for the enrollment were adopted along studies. Stridor (up to 100% [18]) or apnea (up to 60%; [15]) or barking (up to 100% [10]) or chronic cough (up to 75% [15]) were more frequently reported, while less cases had RRI as the main manifestation (up to 56% [15]). The most prevalent diagnoses at admission were asthma (14% [11]) or atopy (18% [10]) or laryngomalacia (7% [10]), yet the diagnosis at admission was available only in 42% of the studies. In 2 retrospective studies, 100% of the patients were admitted because of “symptomatic compression of the trachea or esophagus” [19]. The most used diagnostic procedures were chest imaging studies (including conventional X-ray and/or CT and/or magnetic resonance imaging (MRI) and/or angiography; 90% of the studies), bronchoscopy (80% of the studies) and esophagography (70% of the studies, with apnea and/or or gastrointestinal complaints such as dysphagia or suspected gastroesophageal reflux as main indications). It is worth noting that, when the indication was the “symptomatic compression of the trachea or esophagus”, the preferred diagnostic approach usually combined chest imaging studies, esophagography and bronchoscopy [12, 19]. Based on the results of the diagnostic work-up, the most prevalent final diagnoses were AIA (from 18 to 100% of the cases) or double aortic arch (from 2 to 54% of the cases) or right aortic arch (from 3 to 31% of the cases). Data on treatment (available in 18 of 20 studies) showed that the approach was solely operative in 9/20 studies (45%) or mixed (*i.e.*, operative, and conservative) in the remaining 11 (55%). In 13/20 studies, in which the most prevalent cause of tracheal compression was AIA [20], surgery was adopted in a highly variable proportion of cases (14% to 100%), whereas the conservative approach (with symptomatic medical treatment and observation on an out-patient basis) was undertaken in less cases but with the same variable proportion (3% to 86%). Overall, indication for surgery (available in all except 5 studies) was the persistence of moderate to-severe symptoms (such as apnea and/or > 2 episodes of tracheobronchitis or pneumonia and/or severe stridor and/or cyanosis and/or dyspnea and/or failure to thrive) that were judged unresponsive to conservative treatment. A tracheal compression > 50% was the main reason for deciding surgery only in one study [18]. Overall, data on long-term follow-up of patients undergoing surgical correction of AIA showed complete resolution of symptoms in a proportion of cases ranging from 40 to 100%, while less cases showed persistence of symptoms [21]. Postsurgical complications, including pericardial effusion or pneumonia or surgical wound infection, were rarely reported and all recovered after causal treatment [11]. Conversely, data on the conservative approach indicated that improvement or remission of symptoms occurred slowly, after different period (up to 14 months). Importantly, the observation of improvements depended not only on the degree of TC, but also on the severity, duration and number of cough episodes before diagnosis, as well as on comorbidities [10].

**Table 2:** Main findings from studies on vascular anomalies including aberrant innominate artery

Author [Reference] N of cases	Age	Presenting features (% of cases)	Diagnosis at admission (%)	Diagnostic procedures	Final diagnosis (%)	Therapeutic approach (%)	Indications for surgery	Long-term follow-up (%)
**Fearon** [17] **104 cases**	0–11 yrs	Cyanosis Stridor Apnea	Tracheoesophageal fistula Thymus enlargement Laryngomalacia Cystic fibrosis Hiatus hernia Foreign body Asthma; RRI	Esophagography Bronchoscopy Chest x-ray Angiogram	AIA (66%) DAA (11%) RAA (3%) PAS (3%) ARSA (2%) Others (7%) Unknown (8%)	Operative Conservative	NA	NA
**Mustard** [20] **285 cases**	0–3 yrs	NA	NA	NA	AIA (100%)	Operative (14%) Conservative (86%)	Apnea and/or > 2 episodes of tracheabronchitis or pneumonia	Resolution (60%)^a^ or persistence of symptoms (25%)^a^ Poor outcome (15%)^a^
**Eklof** [21] **30 cases**	0–5 yrs	Stridor; Wheezing Belly cough Hoarse voice Respiratory distress RRI; Dysphagia Cyanosis	NA	Esophagography Chest x-ray Angiogram	AIA (13%) DAA (47%) RAA (17%) ARSA (13%) PAS (10%)	Operative (97%) Conservative (3%)	Stridor RRI Cyanosis Dysphagia	Complete (40%) or partial resolution (20%) Persistence of symptoms (13%) Death (27%)
**Moes** [27] **90 cases**	NA	Stridor (83%) Apnea (26%) RRI (32%)	NA	Esophagography Tracheography Bronchoscopy Chest x-ray Angiogram	AIA (100%)	Operative (67%) Conservative (33%)	Apnea and/or respiratory distress with severe tracheal narrowing	Complete resolution (57%)^a^ partial resolution (28%) or persistence of symptoms (15%)^a^
**Welz** [26] **16 cases**	0–1 yrs	Stridor (75%) RRI (50%) Apnea (44%)	NA	Tracheography Bronchoscopy Angiograms	AIA (100%)	Operative (37.5%) Conservative (62.5%)	Apnea	Resolution of symptoms (100%)
**Marmon** [14] **54 cases**	0–10 yrs	Dysphagia Wheezing Stridor Apnea RRI	NA	Esophagography Bronchoscopy Angiogram	AIA (18%) DAA (44%) RAA (31%) PAS (5%)	Operative (100%)	NA	Resolution (87%) or persistence of symptoms (2%) Death (2%) Lost on follow up (9%)
**Strife** [28] **936 cases**	0–17 yrs	Normal population (n = 807) Congenital heart disease (n = 129)	NA	Chest X-ray Angiogram	AIA (30%)^b^	NA	NA	NA
**Ardito** [29] **78 cases**	0–8 yrs	Apnea (36%) Stridor (32%) Cough (14%) RRI (14%)	NA	Esophagography Bronchoscopy Chest X-ray Angiogram	AIA (100%)	Operative (42%) Conservative (58%)	Apnea	Resolution (85%) or persistence of symptoms (15%)^a^
**Hawkins** [25] **29 cases**	0–15 yrs	Apnea (59%) Stridor (24%) RRI (14%) Exercise-asthma and stridor (3%)	NA	Esophagography Bronchoscopy Chest MRI Chest CT	AIA (100%)	Operative (100%)	Apnea and/or > 2 tracheobronchitis or pneumonia and/or severe stridor	Resolution (93%) or persistence of symptoms (7%)
**Anand** [30] **41 cases**	0–3 yrs	NA	NA	NA	AIA (19%) DAA (44%) RAA (27%) PAS (10%)	Operative (100%)	NA	Resolution (70%) or persistence of symptoms (30%)
**Adler** [18] **25 cases**	0–8 yrs	Stridor (100%) Apnea (32%) RRI (28%) Cyanosis (24%)	Asthma (24%)	Esophagography Bronchoscopy Chest x-ray Angiogram	AIA (100%)	Operative (100%)	Tracheal compression > 50% Stridor apnea Recurrent pneumonia Uncontrolled asthma	Resolution (96%) or persistence of symptoms (4%)
**Jones** [16] **12 cases**	0–3 yrs	Stridor (100%) Feeding troubles (75%) Cyanosis (25%) Apnea (8%) Respiratory arrest (33%)	NA	Esophagography Bronchoscopy Chest x-ray Chest CT	AIA (100%)	Operative (100%)	Cyanosis Apnea Feeding troubles	Resolution (100%)
**Erwin** [23] **45 cases**	0–11 yrs	Stridor (73%) Apnea (47%) Bark cough (31%) Retractions (24%) RRI (20%) Airway anomaly (20%) Dysphagia (16%)	Tracheoesophageal fistula (16%) Asthma (4%) Subglottic stenosis (2%)	Esophagography Bronchoscopy Angiogram Chest MRI	AIA (75%) LAV (22%) ARSA (2%)	Operative (100%)	Moderate tosevere symptoms	Resolution (87%) or persistence of symptoms (9%) Death (2%) Tracheotomy (2%)
**McLaughling** [19] **35 cases**	0–17 yrs	Stridor or wheezing (100%) RRI (47%) Apnea (20%) Dysphagia (14%) Chronic cough (14%) Failure to thrive (11%) Aspiration pneumonia (6%) Vomiting (6%)	DiGeorge syndrome (3%) VATER (3%) Pectus excavatum (3%) Subglottic hemangioma (3%) Atrial septal defect (3%) Incarcerated hernia (3%) Gastroesophageal reflux (3%) Down syndrome (3%) Cerebral palsy (3%)	Esophagography Bronchoscopy Chest x-ray Chest MRI Chest CT	AIA (9%) DAA (54%) RAA (31%) LAV (3%) PAS (3%)	Operative (100%)	NA	Resolution (71%) or persistence of symptoms (20%) Lost on follow-up (9%)
**Gormley** [15] **16 cases**	0–10 yrs	Stridor (100%) Chronic cough (75%) Dyspnea (75%) Apnea (60%) RRI (56%) Dysphagia (25%)	Laryngomalacia (12%) Asthma (12%) Recurrent croup (12%) Sleep apnea (6%)	Esophagography Bronchoscopy Fluoroscopy Chest X-ray Angiogram Chest CT	AIA (94%) DAA (6%)	Operative (75%) Conservative (25%)	Apnea Exercise intolerance RRI unresponsive to treatment	Resolution of symptoms (75%) Mild/residual stridor (25%)
**Woods** [12] **82 cases**	0–12 yrs	Stridor (46%) RRI (35%) Feeding difficulty (15%)	Suspected tracheoesophageal compression	Esophagography Bronchoscopy Chest X-ray Angiogram Chest MRI Chest CT	AIA (24%) DDA (38%) RAA (27%) ARSA (5%) PAS (4%) ALSA (2%)	Operative (100%)	Persistent symptoms of tracheo esophageal compression	Complete resolution (70%) partial resolution (19%) or symptoms of complications (11%)
**Malik** [22] **29 cases**	0–5 yrs	Stridor (69%) Cyanosis (31%) Apnea (21%) Choking episodes (14%) RRI (7%)	NA	Esophagography Bronchoscopy Angiogram Chest MRI	AIA (38%) DAA (10%) RAA (7%) Others (17.5%) None (27.5%)	Operative (24%) Conservative (76%)	Severe or complicated# cases	NA
**Grimmer** [24] **22 cases**	0–7 yrs	Stridor (86%) Cyanosis (50%) Apnea (41%) Cough (41%) Intubation (9%) Failure to thrive (9%) Dysphagia (9%) Ventilator need (4%)	NA	Bronchoscopy Angiogram Chest MRI Chest CT	AIA (100%)	Operative (100%)	Apnea Cyanosis Dyspnea Failure to thrive Oxygen/ Ventilator dependence	Complete/partial resolution (95%) or persistence of symptoms (5%)
**Gardella** [11] **28 cases**	0–13 yrs	Apnea (53%) RRI (50%) Chronic cough (46%) Dyspnea (32%) Stridor (21%) Wheezing (14%)	GER (35%) Overweight (21%) Atopy (14%) Asthma (14%) Laryngomalacia (7%) Emotional paroxysm (3%)	Esophagography Bronchoscopy Chest MRI Chest CT	AIA (100%)	Operative (57%) Conservative (43%)	Severe symptoms, also including QoL No improvement after conservative treatment	Resolution of symptoms (100%)
**Ghezzi** [10] **209 cases**	6.4 yrs	Barking cough (100%) RRI (20%) Exercise induced cough (17%) Dysphagia (6%) Stridor (4%)^c^	Bronchial obstruction (14%) Atopy (18%) GER disease (22%)^c^	Bronchoscopy Chest CT	AIA (25%) RAA (4%) DAA (2%) ARSA (1%) None (67%)	Operative (20%) Conservative (80%)^c^	Severe symptoms, also including QoL No improvement after conservative treatment^d^	Faster improvement of symptoms in the operative *versus* the conservative group^d^

*Abbreviations*: *N* number, *Yrs* years, *RRI* Recurrent respiratory tract infections, *AIA* Anomalous Innominate Artery, *DAA* Double Aortic Arch, *RRA* Right Aortic Arch, *PAS* Pulmonary Artery Sling, *ARSA* Aberrant Right Subclavian Artery, *NA* Not Available, *CVR* Complete Vascular Ring, *VATER* Vertebral defects, Anus defects, Tracheoesophageal fistula, and Radial and Renal dysplasia, *ALSA* Aberrant Left Subclavian Artery, *GER* gastroesophageal reflux, *LAV* Left Arch Variant, *MRI* magnetic resonance imaging, *CT* computed tomography, *QoL* quality of life

^a^Percentage referred to surgery group

^b^Percentage referred to the group of children younger than 2 yrs (*n* = 508)

^c^Percentage referred to 68 cases with evidence of tracheal compression

^d^Referred to anomalous innominate artery group

## Discussion

It has been reported that the most severe forms of TC caused by AIA are usually reported in infants with stridor or episodic apnea or even “near-miss” life-threatening events [10]. Older children with less severe symptoms may be identified late or remain undiagnosed, when persistent unexplained barking cough and RRI, often wrongly treated, lead to re-consider the case and the diagnostic work-up [8]. However, determining which subject should undergo invasive diagnostic procedures for confirming AIA is a challenging task, thus hampering the recognition of approximately 2/3 of the affected cases [31]. Finally, once TC from AIA is demonstrated, patients should be addressed to either a surgical procedure or a conservative treatment, a hard-to-take decision, ideally assessed by a multidisciplinary team, including pediatric pulmonologists, chest radiologists, bronchoscopists and cardiac surgeons.

Starting from these considerations, we retrospectively evaluated a small cohort of children with unexplained persistent respiratory symptoms who eventually received an AIA diagnosis. Several findings from the current case series deserve further comments. In our AIA patients, the age of symptoms onset was significantly lower than the age at diagnosis (1.5 *versus* 6 years), thus confirming a large diagnostic delay as previously reported [10]. Yet, all patients underwent several investigations to rule out the most common causes of chronic cough and/or recurrent pneumonia, such as PCD [32], CF and PID [33], and were prescribed multiple medical therapies, which were only partially effective (if not completely useless). Three patients had been previously diagnosed as asthmatics and received prolonged antiasthma medications, mainly ICS [34], which ultimately proved to be ineffective.

As known, the delay in establishing treatment of TC indeed increases the risk of long-term lung obstructive disease [35]. Moreover, while in normal subjects a small number of pathogens may invade the airways without causing a local colonization, subjects with extrinsic airways compression have an increased mucus production and impaired mucociliary clearance, which in turn favor bacteria airway colonization [36]. This may lead to recurrent lung infectious exacerbations and secondary segmental-to-lobar atelectasis. If the airways are not cleared of obstructing mucus by removing the primary cause (in our cases TC from AIA), then a vicious circle of persistent airway obstruction and bacterial airways colonization sets up, thus increasing the risk of protracted bacterial bronchitis and bronchiectasis [8]. In these circumstances, lobar collapse associated with hypoventilation and impaired gas exchange may develop [37]. All the above events occurred in at least 3 current patients with AIA who had marked TC associated with recurrent pneumonia and lobar atelectasis. Finally, frequent (and expensive) investigations have an impact on the costs sustained by the health care system, as well as the repeated (and often non definitive) referral to care centers may increase the psychological burden to families experiencing AIA cases [38].

Over the years, literature has highlighted many controversies about the choice of the diagnostic procedures for confirming an AIA diagnosis. AE is considered by several authors the best means of showing a narrowed trachea and a pulsatile compression on its wall from outside [10, 11]. The endoscopic diagnosis of TC and an estimation of tracheal stenosis severity is based on subjective evaluation by the bronchoscopist during the procedure. Patients undergoing AE should be breathing spontaneously for proper assessment of trachea dynamics, but deep sedation or general anesthesia is often necessary [39], thus requiring a positive-pressure ventilation. The latter might contribute to a missed diagnosis of airway collapse at the time of AE procedure [40]. Tracheal narrowing and tracheomalacia can be indeed evaluated by a skilled AE team if the patient is slightly sedated or in the awakening stage of the procedure, when cough reflex finds out trachea collapse [10]. This represents a potential, albeit relevant, drawback of relying only on the AE for the assessment of an AIA condition, since AE can record only the existing pulsatile compression on the tracheal wall. Conversely, chest imaging with contrast medium, including MRI and CT (or CT angiography), are effective modalities to identify the vessel compressing the airways and quantify tracheal stenosis by standardized measurement [35]. They both have advantages and disadvantages. Chest MRI effectively images and accurately characterizes the thoracic vascular abnormalities and serves to exclude AIA-mimicking conditions, such as other vascular disorders, mediastinal masses, intrinsic upper airways or upper gastrointestinal tract abnormalities [41]. Despite the long acquisition time and the need that patients are either cooperative or sedated, MRI avoids radiation exposure and is ideal when follow-up imaging is required [42]. Regrettably, the equipment is not universally available, and results require expertise in interpretation. Nevertheless, in case of AIA chest MRI is also used as a pre-operation procedure, either to define accurately the vascular anatomy or plan the surgical intervention [19, 22]. Chest CT is recognized as the gold standard modality for demonstrating densities due to lobar-to-segmental pneumonia or atelectasis or interstitial disease or malformations or bronchiectasis [22, 42, 43] or the pulmonary vascular structures (the latter best visualized by CT angiography) [44]. Compared to MRI, CT is cheaper and almost universally available, and provides high-spatial resolution images with fast acquisition times. Exposure to ionizing radiation is still a matter of concern in pediatric patients, but newer CT equipment, even using angiography, allows for significant radiation dose reduction [44]. Given that all current patients (except for one) had also recurrent pneumonia, obtaining details of lung parenchyma and vessel anatomy was mandatory. The choice of chest imaging (whether CT or MRI) should be tailored to the individual patient, accounting for clinical circumstances, parental/clinician preferences, need for and risks of sedation, imaging equipment and expertise available. Chest images at MRI or CT / CT angiography can be later reviewed and measured to determine the relationship between the trachea and the innominate artery. Difficulties in differentiating a TC due to AIA from a TC due to external masses using AE have been reported [23], unless an obvious vessel-caused extrinsic pulsatile compression is clearly visualized on the trachea. For this reason, AE is usually followed by chest imaging to confirm the AE findings [11, 16, 19, 24, 25]. In cooperating subjects, the flow-volume loop is an additional and effective method to document affected airways in suspected TC patients [22].

A relevant point to be discussed is the choice of treatment of AIA cases. Of the current series, 4 patients underwent surgery as all experienced serious unremitting events, namely chronic dry cough, recurrent pneumonia, lobar atelectasis and spontaneous pneumomediastinum and subcutaneous emphysema (the latter only in case 4), with monthly exacerbations and very short intercritical symptom-free periods. These findings, combined with the demonstration of TC > 50% at CT, led to conclude that surgery was mandatory. Conversely, in 55% of subjects (cases 5 to 9) surgery was not deemed necessary, given the non-severity of the clinical course. Even though symptoms started early, these patients progressively improved, did not report complications and had less infectious exacerbations, fewer episodes of barking cough and progressive extension of the well-being period, as case 9 report has shown. These findings, combined with TC < 50% at chest imaging, led to a conservative management decision. Follow-up confirmed that the clinical course is currently uneventful. Even though patients from previous studies have been addressed either to surgery or conservative treatment, in presence of severe symptoms due to high-grade tracheal stenosis, or if the symptoms do not regress upon medical treatment, surgical treatment is always recommended.

In our case series, surgical interventions consisted of aortopexy (cases 1 and 3) or tracheopexy (cases 2 and 4). Aortopexy is considered the preferred approach in patients with AIA [5, 45]. For aortopexy, pledgeted non resorbable sutures are placed in the adventitia of the ventral surface of the aortic arch without entering the aortic lumen at the origin of the innominate artery. Sutures are placed transternally and transcartilaginously, and tightened and secured to achieve anterior displacement of the aortic arch and the innominate artery [45]. In 2 children with AIA, the major contribution to airway compression was the hypermobility of the *pars membranacea* protruding into the tracheal lumen during cough, as shown by AE. In these cases, posterior tracheopexy consisting of suture of the posterior tracheal membrane to the anterior longitudinal ligament of the spine through a posterior right thoracotomy was considered necessary [46].

**Fig. 3:**
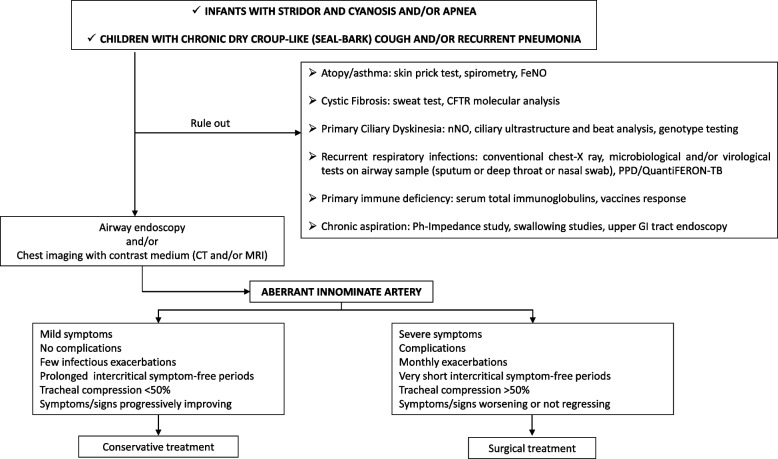
Proposal of management algorithm for infants and children with respiratory symptoms suspected to be secondary to aberrant innominate artery

We retrospectively described a small cohort of children with chronic respiratory disorders which were ultimately attributed TC from AIA and managed through a multidisciplinary approach. We also provided a review of the relevant literature on the topic. Based on current findings, we believe that the diagnostic work up in cases presenting with symptoms or signs of airway compression must include a list of procedures aimed at confirming the suspected abnormality and defining the best therapeutic option as early as possible. We propose a management algorithm which may be helpful for clinicians dealing with infants and children with respiratory symptoms suspected to be secondary to AIA (Fig. 3).

## Conclusions

TC caused by AIA may be a serious cause of chronic respiratory disease in childhood. Early diagnosis and prompt decision of treatment can reduce the risk of long-term airway obstructive disease and improve patients’ daily life. Data from this report may help in addressing the diagnostic work-up and the choice of treatment. A management algorithm of patients suspected of AIA based on the evidence from literature review is proposed. Like all algorithms, it is not meant to replace clinical judgment, but it should rather drive physicians to adopt a systematic approach to the disease.

## Data Availability

The datasets presented in this study are available from the corresponding author upon reasonable request.

## Abbreviations

TC: Tracheal compression
AIA: Aberrant innominate artery
CT: Computed tomography
RRI: Recurrent respiratory infections
CF: Cystic fibrosis
PCD: Primary ciliary dyskinesia
PID: Primary immunodeficiency
ICS: Inhaled corticosteroids
MRI: Magnetic resonance imaging
